# Xanthogranulomatous osteomyelitis of the jaw in a young boy: a case report

**DOI:** 10.1186/s13023-025-04010-w

**Published:** 2025-09-24

**Authors:** Kevin Bloch-Maier, Sophie-Myriam Dridi, Arek Sulukdjian, Anne-Laure Ejeil

**Affiliations:** 1 Department of Maxillofacial Surgery, Beaujon hospital, AP-HP, Paris, France; 2 Department of Periodontology, Institute of Oral Medicine Riquier, CHU Nice, Nice, France; 3 Department of Oral Surgery, Bretonneau Hospital AP-HP, Paris, France; 4 Department of Odontology, Bretonneau Hospital AP-HP, Paris, France

**Keywords:** Xanthogranulomatous osteomyelitis, Pediatric xanthogranulomatous osteomyelitis 2, Oral xanthogranulomatous osteomyelitis 3, Head and neck xanthogranulomatous osteomyelitis 4

## Abstract

Xanthogranulomatous inflammation is a rare chronic inflammatory condition. It has been described in the long bones, and can affect various organs, including the salivary glands, gallbladder, kidneys, and gastrointestinal tract. The diagnosis is made throught histopathology which reveales foamy macrophages alongside polymorphonuclear leukocytes, plasma cells, and polyclonal lymphocytes arranged in a mosaic-like pattern. Here, we present the case of a 16-year-old boy in good general health who was referred by his orthodontist to the oral surgery department at Bretonneau Hospital for an asymptomatic multipartite image of the left mandibular angle that was discovered incidentally on a panoramic radiograph. The clinical examination was unremarkable. Cone beam computed tomography revealed a multipartite osteolytic lesion of the left mandibular angle, but this that was not specific enough for a diagnosis. Magnetic resonance imaging ruled out a vascular malformation. A biopsy revealed xanthogranulomatous osteomyelitis. In summary, this is the second case of xanthogranulomatous osteomyelitis localised to the mandible. This highlights a crucial point : radiological images of xanthogranulomatous osteomyelitis do not allow for a diagnosis; a biopsy is essential to rule out an aggressive or a malignant tumor.

**Supplementary Information:**

The online version contains supplementary material available at 10.1186/s13023-025-04010-w.

## Introduction

Xanthogranulomatous inflammation is a rare chronic inflammatory process [1]. In a recent review, Al Saleem et al. [2] reported only 35 documented cases between 1912 and 2023. It has been observed in the long bones, which are rarely affect [3] and, less commonly, in more than one organ, including the salivary glands, gallbladder, kidneys, and gastrointestinal tract [4].

The main symptoms of bone involvement are pain [3], fracture [5], and swelling [6]. Radiologically, XO primarily manifests as an osteolytic lesion accompanied by a periosteal reaction [3].

A histopathologicaldiagnosis is made with the presence of foamy macrophages alongside with polymorphonuclear leukocytes, plasma cells, and polyclonal lymphocytes in a mosaic-like pattern [7, 8]. A histopathological diagnosis is essential to differentiate it from neoplasm [9].

To our knowledge, this is the second case of mandibular xanthogranulomatous osteomyelitis reported to the jawbone.

## Case presentation

**Fig. 1:**
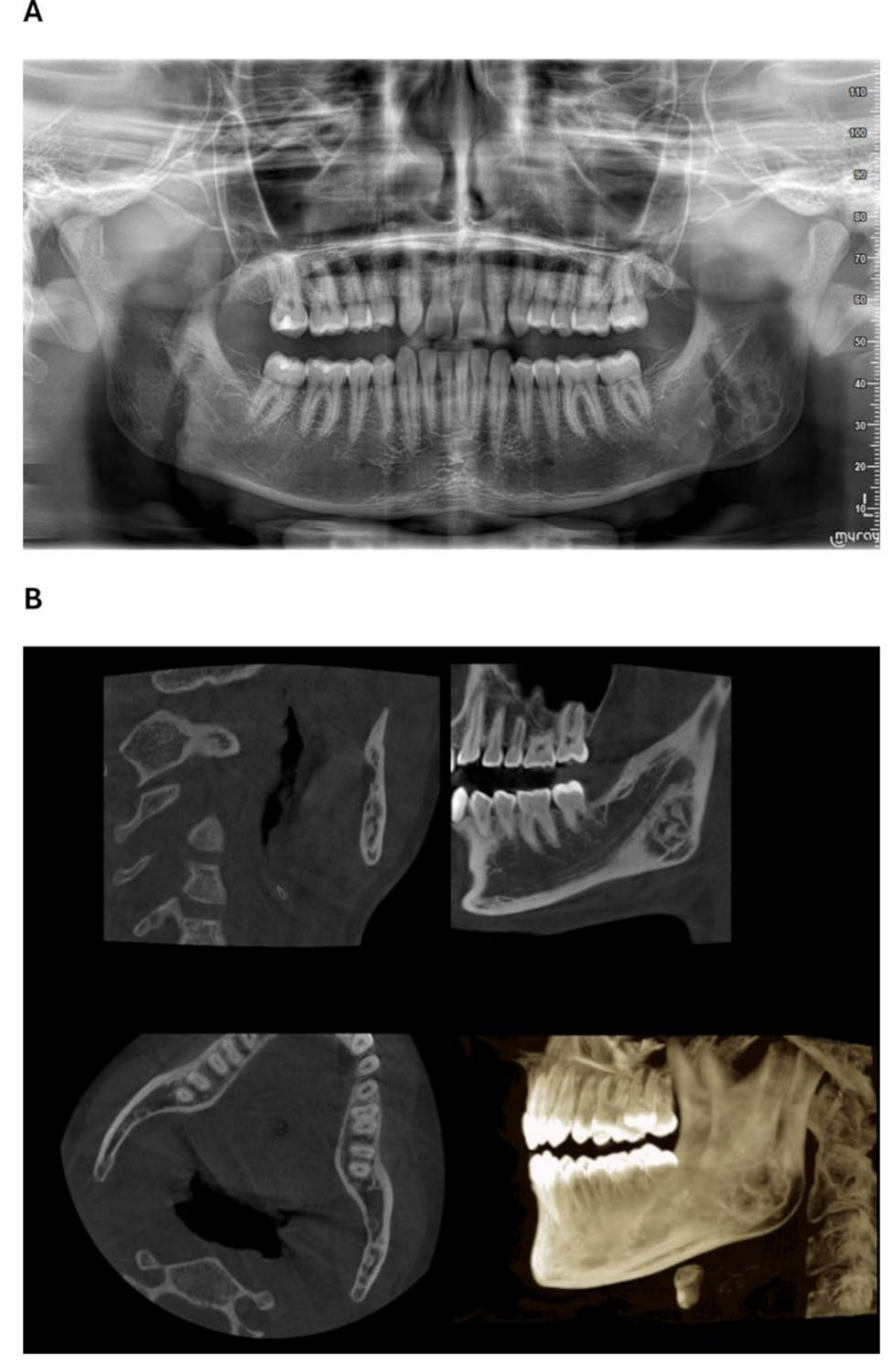
**A**: Initial panoramic radiograph showing an osteolytic lesion of the left mandibular angle. **B**: Initial cone beam computed tomography images: multipartitioned osteolytic lesion of the left mandibular angle

A 16-year-old young man in good general health who reported only a fall at school six years ago with no notable consequences was referred by his orthodontist to the Oral Surgery Department at Bretonneau Hospital for an asymptomatic multipartitioned image of the left mandibular angle discovered incidentally on a panoramic radiograph (Fig. 1A).

Extraoral examination revealed no cervical lymphadenopathy or facial asymmetry. Intraoral examination did not reveal any cortical swelling, and the oral mucosa was unremarkable.

The diagnostic process was initiated with cone beam computed tomography (CBCT). The images revealed a multipartitioned osteolytic lesion of the left mandibular angle that lacked specificity for a diagnosis (Fig. 1B). An overall thickening of the ascending branch was identified in comparison with the contralateral ascending branch.

Magnetic Subsequently, magnetic resonance imaging (MRI) was performed. It highlighted a lesion of the left mandibular angle, located posterieur to the inferior dental nerve, in the T1 and T2 hyposignals, exhibiting heterogeneous uptake of contrast agent following gadolinium injection. The lesion measured 14 mm × 3 mm × 13 mm. The diagnoses suggested by the radiologist are fibrous dysplasia or desmoplastic fibroma, and a vascular malformation is excluded.

In the context of an osteolytic lesion of the mandibular angle, several other diagnostic hypotheses are considered, such as ameloblastoma, a keratocyst, an essential cyst, Ewing sarcoma, or osteosarcoma.

**Fig. 2:**
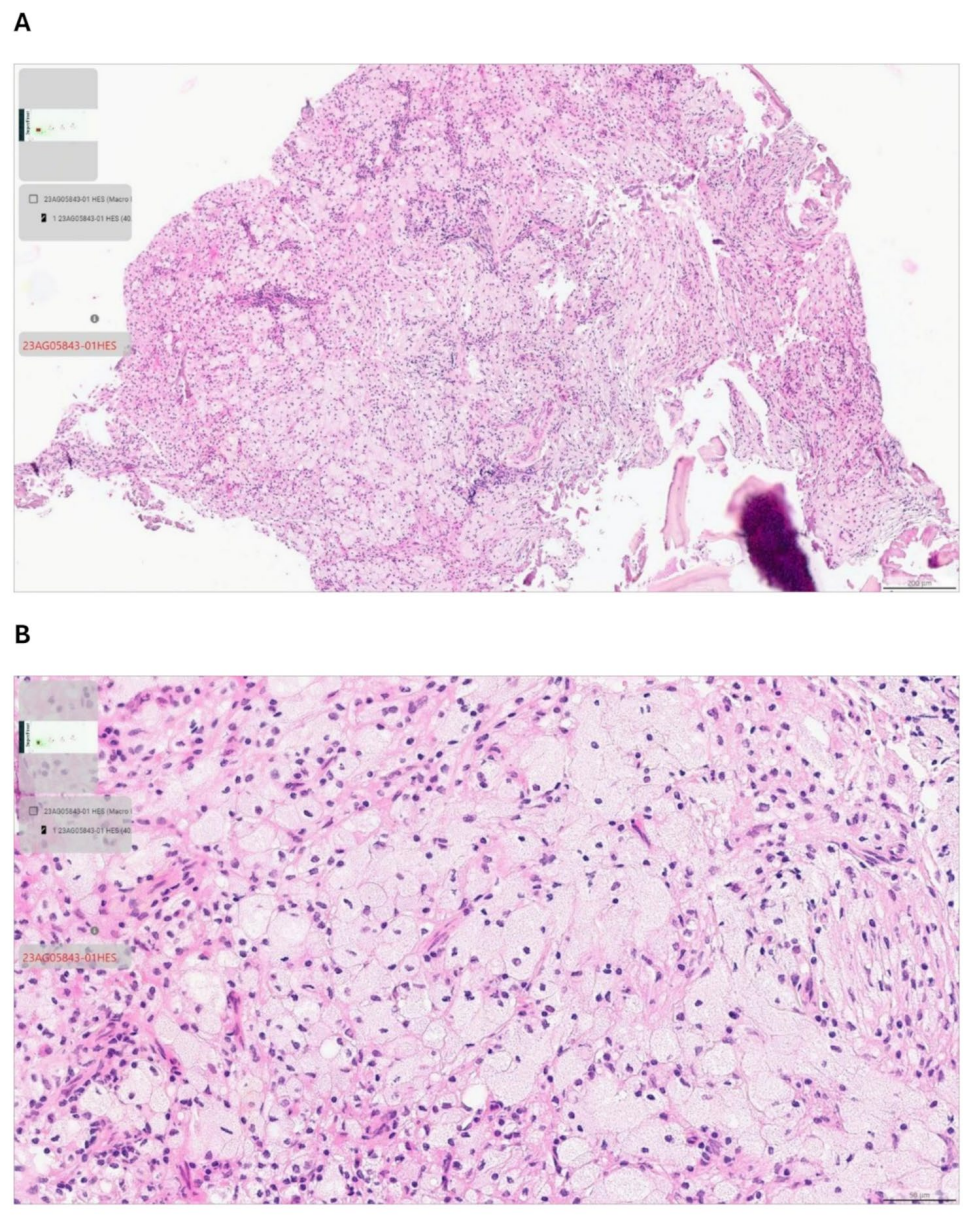
Imaging and microscopic features: Hematoxylin and eosin 7 × (**A**) and 27 × (**B**) magnification histopathological images reveal inflammatory infiltration characterized by the presence of lymphocytes, interspersed with foamy macrophages

Due to the patient’s age and the location of the tumor, an incisional biopsy was performed under general anaesthesia. The findings revealed an inflammatory reaction with numerous foamy macrophages mixed with lymphocytic cells (Fig. 2A and B), thus leading to the diagnosis of xanthogranulomatous osteomyelitis (XO). The postoperative course was uneventful, and the patient was discharged from hospital after 24 h. The prescription given to the patient included amoxicillin and clavulanate (1 g/125 mg) for 2 days, paracetamol 1 g 3 times a day, Prednisolone 60 mg and 0.12% chlorhexidine mouthwash for 15 days to reduce inflammation and pain and to prevent postoperative infection.

A follow-up review was conducted with the patient two weeks after the intervention. The results of the clinical examination were found to be entirely normal.

Following deliberation by the clinical staff and consultation with parents regarding the benefit/risk balance, the decision was taken against performing surgical revision and instead to establish clinical and radiological monitoring. We scheduled follow-up visits at three months, six months, and then every year. Follow-up visits were scheduled at three-month intervals, with subsequent visits conducted annually.

**Fig. 3:**
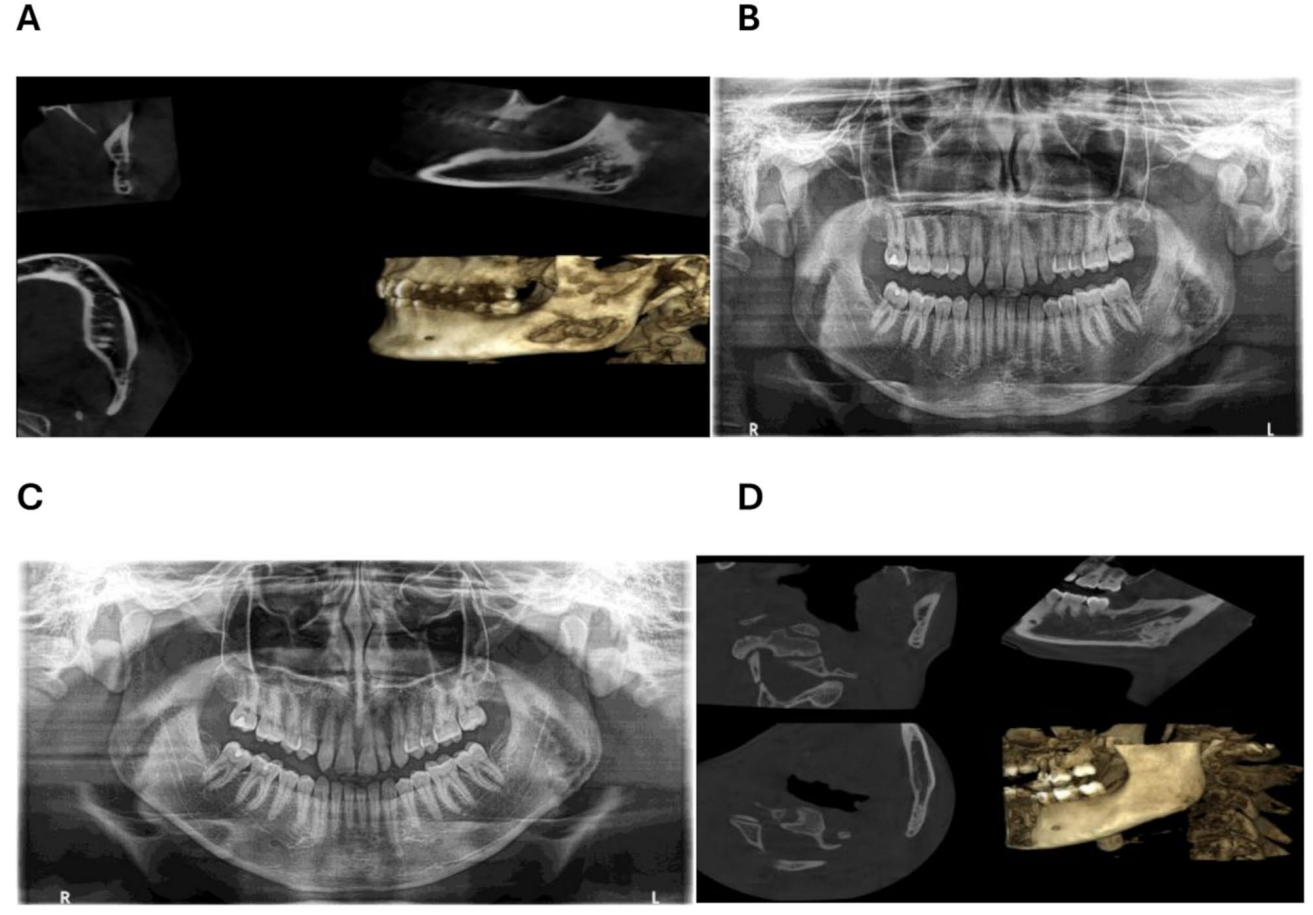
**A**: CBCT at 3 months. **B**: Panoramic radiography at 6 months. **C**: Panoramic radiography at one year. **D**: CBCT at one year

Three months following the biopsy, the control CBCT image revealed a reossification of the biopsied area (Fig. 3A). AT the six-month mark, a comprehensive radiographic analysis was conducted, revealing no discernible progression of the lesion (Fig. 3B). At one year, the patient underwent a panoramic radiography (Fig. 3C) and a CBCT (Fig. 3D), both of which revealed regression of the lesion.

## Discussion

XO is a rare chronic inflammatory bone disease characteried by the accumulation of cholesterol-laden foamy macrophages, histiocytes, and plasma cells. It is generally considered to be a benign condition that is treatable. Bone involvement is less common prevalent and is primarily observed in soft tissues. Indeedthe prevalence of this condition is highest in the kidney, followed by the gallbladder [10]. The lung, brain and bone are rarely affected [9, 11, 12]. To the best of our knowledge, only one case of jaw involvement has been described in the literature [13].

In cases involving bone involvement, radiological examination often reveals lytic lesions in the majority of patients, as evidenced by our patient’s case.

In order to establish a diagnosis of XO, it is necessary to correlate the clinical, radiological and histopathological findings. However, histopathology remains the gold standard for the final diagnosis of this disease [14].

The histopathological mechanisms underlying xanthogranulomatous osteomyelitis are complex, involving several inflammatory and cellular processes. The precise pathogenesis of this condition remains to be elucidated; however, it can be hypothesised that the disease’s hallmarks of chronic inflammation, granuloma formation and accumulation of xanthomatous cells may ultimately result in bone destruction [1].

As demonstrated in previousstudies, XO has been shown to be associated with a delayed-type hypersensitivity reaction involving cell-mediated immunity [4] or trauma [2]. In this particular instance, the young patient reported experiencing head trauma without loss of consciousness or injury following a fall at school six years previously.

To date, no effective treatment approach for XO has yet been established. Most lesions are managed with surgical excision in order to differentiate them from malignancies [8] associated with antibiotics. In the event of an infection is being detected by bacteriological sampling, a mere three authors recommend treatment with antibiotics, whilst also advocating and long-term and regular follow-up prior to resorting to surgery [2–4]. In 2019, Solooki et al. [3] recommended the use of broad-spectrum antibiotics prior to excisional surgery. The patient was admitted to hospital on the day of the incisional biopsy procedure and discharged 24 h after the surgical intervention. The administration of antibiotic prophylaxis was initiated exclusively for the purpose of regulating the surgical procedure and averting any potential postoperative infection.

In the majority of cases, excisional surgery is performed to rule out malignancy or extensive infection [3, 6, 10, 15, 16].

In this case, given the absence of any detected infectious germs, and in consideration of the inconclusive findings regarding the efficacy of antibiotic therapy as an alternative to surgical intervention, a collective decision was reached, with the consent of the parents, to forgo surgical intervention, which was deemed to carry a high risk of damaging the inferior alveolar nerve. Instead, a course of close clinical and radiological monitoring was initiated. This approach diverged from the management strategy employed by Baeh et al. [13], who opted for a surgical intervention, despite the potential risks [13].

To the best of our knowledge, this is the second report of XO occurring in the jaw. The majority of reported cases have been found to occur in long bones.

It is imperative that further studies must be conducted in order to determine the most appropriate therapeutic management strategies.

## Conclusion

In summary, we present here the second case of XO with mandibular localization. This underscores a crucial challenge: radiological imaging of XO does not permit diagnosis; a biopsy is imperative to exclude the possibility of an aggressive tumor or a malignant tumor.

Consequently, clinicians and radiologists should be cognisant of thepotential for XO development at the mandibular level and include it into their differential diagnoses.

The present study has enabled the demonstration of complete healing of the bone lesion following a 2-year follow-up of the patient. This outcome demonstrates the efficacy of close monitoring in avoiding surgical intervention and the serious consequences thereof, particularly in children.

## Supplementary Information

Below is the link to the electronic supplementary material.

Supplementary Material 1

## Data Availability

The datasets used and/or analyzed during the current study are available from the corresponding author on reasonable request.

## Abbreviations

CBCT: Cone beam computed tomography
XO: Xanthogranulomatous osteomyelitis
MRI: Magnetic resonance imaging
