# Correction to: Xanthogranulomatous osteomyelitis of the jaw in a young boy: a case report

**DOI:** 10.1186/s13023-026-04268-8

**Published:** 2026-02-19

**Authors:** Kevin Bloch-Maier, Sophie-Myriam Dridi, Arek Sulukdjian, Anne-Laure Ejeil

**Affiliations:** 1 Department of Maxillofacial Surgery, Beaujon Hospital, AP-HP, Paris, France; 2 Department of Periodontology, Institute of Oral Medicine Riquier, CHU Nice, Nice, France; 3 Department of Oral Surgery, Bretonneau Hospital AP-HP, Paris, France; 4 Department of Odontology, Bretonneau Hospital AP-HP, Paris, France

**Correction to: Orphanet Journal of Rare Diseases (2025) 20:488**

10.1186/s13023-025-04010-w

Following publication of the original article [1], the authors identified an error in the authors names’ order. The given name and family name were erroneously transposed.

The incorrect author’ names are:

Bloch-Maier Kevin 1,

Dridi Sophie-Myriam 2,

Sulukdjian Arek 3 and

Ejeil Anne-Laure 4*

The correct authors’ names are:

Kevin Bloch-Maier 1,

Sophie-Myriam Dridi 2,

Arek Sulukdjian 3 and

Anne-Laure Ejeil 4*

The author group has been updated above and the original article [1] has been corrected.
